# Macrophage respiratory complex III governs immune evasion

**DOI:** 10.1097/IN9.0000000000000065

**Published:** 2025-07-04

**Authors:** Marie Anne-Catherine Neumann, Christian Frezza

**Affiliations:** 1 Department I of Internal Medicine, Center for Integrated Oncology Aachen Bonn Cologne Duesseldorf (CIO ABCD), Faculty of Medicine and University Hospital of Cologne, University of Cologne, Cologne, Germany; 2 Faculty of Medicine and University Hospital Cologne, Institute for Metabolomics in Ageing, Cluster of Excellence Cellular Stress Responses in Aging-associated Diseases (CECAD), University of Cologne, Cologne, Nordrhein-Westfalen, Germany; 3 University of Cologne Faculty of Mathematics and Natural Sciences, Institute of Genetics, Cluster of Excellence Cellular Stress Responses in Aging-associated Diseases (CECAD), Cologne, Nordrhein-Westfalen, Germany

**Keywords:** mitochondria, metabolism, ROS, inflammation, immune evasion, IL-10

## Abstract

The intricate interplay between cellular metabolism and immune function has emerged as a pivotal area of research in immunology. Macrophages, as central players in the innate immune system, exhibit remarkable metabolic flexibility that influences their activation states and functional outputs, with important implications for the pathophysiology of inflammatory diseases and cancer. A recent study by Zotta and colleagues provides new insights into the role of mitochondrial complex III (CIII) in regulating the anti-inflammatory cytokine interleukin-10 (IL-10) and its implications for tumor immunity.

Macrophages, as key players in innate immunity, exhibit remarkable metabolic plasticity, dynamically shifting between glycolysis and oxidative phosphorylation to adjust their energy metabolism to their activation state. This metabolic reprogramming is not merely a byproduct of activation but a crucial determinant of macrophage functional phenotype. For instance, upon stimulation by signals such as lipopolysaccharide (LPS), macrophages undergo profound metabolic changes that culminate with the production of metabolites, including itaconate, fumarate, and succinate, needed for full-blown activation ^[1,2]^. Therefore, mitochondrial metabolism is increasingly recognized as a central regulator of immune cell function, governing processes that extend beyond energy production ^[3]^. Within the electron transport chain (ETC), respiratory complex III (CIII) plays a key role in maintaining redox balance, modulating cytokine production, and fine-tuning immune cell activation ^[4,5]^. A critical component of this regulation is the controlled generation of reactive oxygen species (ROS), serving as signaling molecules in immune responses. Among the cytokines influenced by ROS, interleukin-10 (IL-10) stands out as a crucial anti-inflammatory mediator, curbing excessive inflammation and promoting tissue repair ^[6–9]^. By modulating macrophage activation states, IL-10 helps balance pro-inflammatory and anti-inflammatory responses, ensuring a coordinated and effective immune defense while preventing pathological inflammation. However, in the context of tumors, IL-10 mostly produced by tumor-associated macrophages (TAMs) has a more complex role. On one hand, elevated IL-10 levels in aggressive cancers, such as melanoma and colorectal cancer, contribute to immune evasion by suppressing cytotoxic T cell activity, thereby promoting tumor progression ^[10,11]^. On the other hand, IL-10 has been reported to enhance the effector function of cytotoxic T cells and natural killer (NK) cells ^[7,12,13]^, reducing tumor growth. While previous research has suggested a connection between mitochondrial metabolism and IL-10 production, the precise molecular mechanisms underlying this relationship and its relevance to cancer remain poorly understood. The study by Zotta and colleagues ^[14]^ investigates the role of mitochondrial CIII in IL-10 production and tumor-mediated immune evasion. The authors employ pharmacological inhibitors, S3QEL 1.2 and myxothiazol, to specifically target CIII and assess their effects on IL-10 production in macrophages. They demonstrate that inhibition of CIII results in a significant reduction of IL-10 levels in LPS-activated macrophages, both in vitro and in vivo. Mechanistically, they identify the transcription factor activator protein 1 (AP-1) and its subunit c-Fos as key mediators of IL-10 expression, with CIII-derived ROS being necessary for AP-1 activation. Notably, in the murine model of B16F10 melanoma, the treatment with S3QEL 1.2 decreases IL-10 production by TAMs, thereby enhancing anti-tumor immunity and reducing melanoma progression in those mice. By elucidating the role of CIII in sustaining IL-10 production, Zotta and colleagues highlight a potential metabolic checkpoint that tumors may exploit to evade immune surveillance. It suggests that targeting mitochondrial CIII-dependent ROS generation could represent a novel strategy for modulating the tumor microenvironment and improving immunotherapy outcomes (Figure 1).

**Figure 1.**
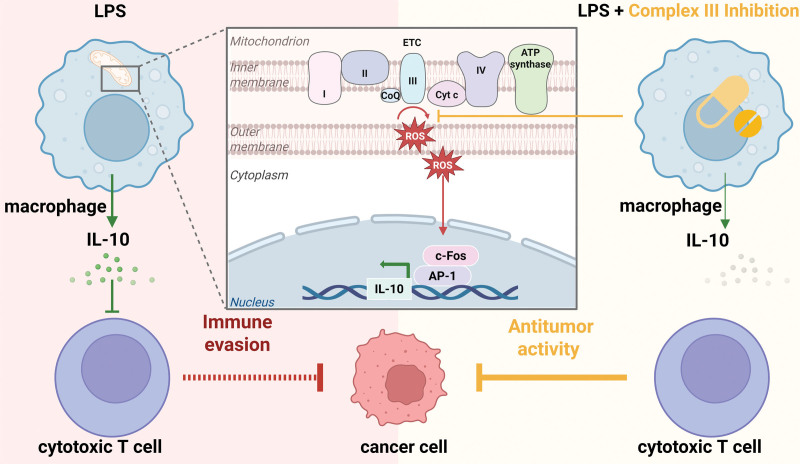
**Identification of complex III (CIII) as metabolic checkpoint.** This schematic illustrates the role of mitochondrial CIII in the regulation of interleukin-10 (IL-10) production in macrophages and its implications for tumor immunity. Upon activation by lipopolysaccharide (LPS), macrophages undergo metabolic reprogramming involving the electron transport chain (ETC), particularly CIII. This process leads to the generation of reactive oxygen species (ROS), which act as signaling molecules to activate the transcription factor complex activated protein 1 (AP-1), with c-Fos as a critical subunit. The AP-1/c-Fos complex promotes transcription of the anti-inflammatory cytokine IL-10. In the tumor microenvironment, tumor-associated macrophages upregulate IL-10, leading to the suppression of cytotoxic T cell activity and promoting immune evasion. Pharmacological inhibition of CIII (eg, with S3QEL 1.2 or myxothiazol) reduces ROS production, thereby impairing AP-1 activation and IL-10 expression. This restores T cell-mediated anti-tumor activity and reduces tumor progression. Figure created in BioRender.com.

While this work paves the way for new strategies to curb immune evasion based on metabolic checkpoints, some questions remain to be addressed. For instance, it is important to note that c-Fos/AP-1 is not specific to IL-10 transcription; it regulates numerous genes involved in proliferation, differentiation, and immune responses. While essential for IL-10 induction here, it likely drives broader transcriptional programs ^[15]^ that could participate in tumor evasion.

Furthermore, the study utilized pharmacological inhibitors such as S3QEL 1.2 and myxothiazol to suppress CIII activity. While these inhibitors have been instrumental in uncovering the role of CIII in IL-10 regulation, their specificity should be assessed. Given that small-molecule inhibitors often exhibit off-target effects ^[16]^, it is crucial to determine whether the observed changes in IL-10 production and macrophage function are exclusively due to CIII inhibition or if other mitochondrial or cellular pathways are involved. Employing genetic approaches, such as CRISPR-mediated deletion or RNA interference, could provide a more precise validation of the specific role of CIII in this process. In addition, mitochondrial respiration is known to be critical for cancer cell growth and survival ^[17]^. It remains unclear whether the anti-tumor effects of S3QEL 1.2 arise solely from immune modulation or also involve direct effects on tumor cell metabolism. While the authors acknowledge the lack of information, they do not present data addressing this point. In vitro experiments on B16F10 melanoma cells alone or a xenograft experiment in immunodeficient mice could rule out a tumor-intrinsic effect. Although the study demonstrated promising short-term effects of CIII inhibition in suppressing tumor-associated macrophage function, the long-term consequences of disrupting mitochondrial metabolism remain largely unexplored. Chronic inhibition of CIII may lead to metabolic adaptations, altered cellular homeostasis, or unintended immune dysregulation in multiple cell types, resulting in tissue dysfunction or affecting systemic immunity. Longitudinal studies assessing metabolic adaptation, immune resilience, and exhaustion, as well as potential toxicity, will be critical for evaluating the feasibility of CIII inhibition as a therapeutic strategy. In this regard, the above-described dual role of IL-10 across immune cell types needs to be evaluated for carefully nuanced therapeutic strategies.

Finally, the research was conducted using murine macrophage models, which, while informative, may not fully recapitulate human immune responses. Differences in mitochondrial metabolism, cytokine regulation, and immune signaling pathways between murine and human macrophages necessitate validation in human cells ^[18,19]^. In the study, experiments using LPS-stimulated macrophages derived from human peripheral blood mononuclear cells demonstrated that IL-10 expression was suppressed due to CIII inhibition, a finding that mirrors the effects observed in murine models. Investigating downstream effects is pending and could provide crucial insights into its clinical relevance.

In summary, this work leverages the specific inhibition of CIII-derived ROS to regulate IL-10 production in macrophages, thereby enhancing tumor immunity. It is part of a larger effort aimed at targeting mitochondrial proteins to modulate cellular metabolism in disease. Small molecules designed to inhibit or modulate ETC components are currently under investigation, with some advancing to phase 1 clinical trials. These compounds hold potential for therapeutic applications in cancer, metabolic disorders, and immune regulation. However, their safety profiles and efficacy remain key concerns, as mitochondrial dysfunction can lead to unintended side effects. Further studies are required to evaluate their tolerability, minimize adverse effects, and optimize their clinical utility.

## Conflict of interest

The authors declare that they have no conflicts of interest.

## Funding

M.A.-C.N. is supported by the Cologne Clinician Scientist Program (CCSP), Faculty of Medicine, University of Cologne, Germany, funded by the Deutsche Forschungsgemeinschaft (DFG, German Research Foundation) (Project No. 413543196) and by the Köln Fortune Program, Faculty of Medicine, University of Cologne, Germany. C.F. is supported by a Cancer Research UK (CRUK) Programme Foundation Award (PFA; C51061/A27453) and by the Alexander von Humboldt Foundation in the framework of an Alexander von Humboldt Professorship endowed by the Federal Ministry of Education and Research.
